# Acute beetroot juice ingestion fails to improve sprint performance and neuromuscular function in trained male sprinters: a randomized, double-blind, placebo-controlled study

**DOI:** 10.1080/15502783.2026.2674220

**Published:** 2026-05-18

**Authors:** Álvaro López-Samanes, Diego Moreno-Pérez, Millán Aguilar-Navarro, Alejandro Muñoz, Miguel López-Moreno, Marta Garcés-Rimón, Ignacio Díez-Vega, Justin Roberts, Juan Del Coso

**Affiliations:** a GICAF Research Group, Education, Research Methods and Evaluation Department, Faculty of Human and Social Sciences, Universidad Pontificia Comillas, Madrid, Spain; b Institute of Health and Sport Sciences, Faculty of Health Science, Universidad Francisco de Vitoria, Madrid, Spain; c Grupo de Investigación en Biotecnología Alimentaria, Universidad Francisco de Vitoria, Madrid, Spain; d Departamento de Enfermería y Fisioterapia, Facultad de Ciencias de la Salud, Universidad de León, León, Spain; e Cambridge Centre for Sport and Exercise Sciences, Faculty of Science and Engineering, Anglia Ruskin University, Cambridge, UK; f Sport Sciences Research Centre, Rey Juan Carlos University, Fuenlabrada, Spain

**Keywords:** Dietary nitrate, nitric oxide, sports performance, sprint athletes, dietary supplements, track and field

## Abstract

**Introduction:**

The aim of this study was to evaluate the acute effect of beetroot juice ingestion on sprint performance and neuromuscular properties of male trained sprinters.

**Methods:**

Twelve male sprinters (24.3 ± 4.8 years) participated in a randomized, double-blind, and placebo-controlled study with two experimental trials after the ingestion of 70 mL beetroot juice (containing 6.4 mmol NO_3_−) or 70 mL placebo drink (containing 0.04 mmol NO_3_−). Participants performed a countermovement jump, a squat jump, and 60 and 100 m sprint tests at 150 minutes after ingestion. Before and after these performance tests, contractile properties of the rectus femoris, biceps femoris, gastrocnemius lateralis, and gastrocnemius medialis muscles were measured with tensiomyography.

**Results:**

Salivary concentrations of NO_3_− and nitrite NO_2_− were also measured and in comparison to the placebo drink, the intake of beetroot juice increased salivary concentrations of NO_3_− (from 230 ± 435 vs 6164 ± 3370 μM; *p* = < 0.001, ES = 1.53) and NO_2_− (130 ± 131 vs 4509 ± 4895 μM; *p* = 0.018; ES = 1.63). Beetroot juice ingestion did not affect countermovement jump height (44.12 ± 6.14 vs 43.6 ± 6.29 cm; *p* = 0.400; ES = −0.25), squat jump height (42.02 ± 4.92 vs 41.91 cm; *p* = 0.911; ES = 0.04), 60 m sprint time (7.55 ± 0.32 vs 7.58 ± 0.32 seconds; *p* = 0.407; ES = −0.24), or 100 m sprint time (12.57 ± 0.65 vs 12.51 ± 0.62 seconds; *p* = 0.343; ES = 0.29). The tensiomyography analysis did not report any difference in the contractile properties of the muscles analyzed (*p* = 0.065–0.914), except for the biceps femoris in the relaxation time variable (*p* = 0.047).

**Conclusion:**

Acute ingestion of 70 mL beetroot juice containing 6.4 mmol of NO_3_− did not enhance sprint performance or the neuromuscular function of male trained sprinters.

Trial registration: The study was retrospectively registered in ClinicalTrials.gov with the following ID: 5-56NCT06675682 by 1 November 2024.

## 1. Introduction

Beetroot juice is a rich source of nitrate (NO_3_−), which serves as a precursor to nitric oxide (NO) via the nitrate (NO_3_−) to nitrite (NO_2_−) to NO pathway [1]. Beetroot juice is recommended for aerobic-dominant events because nitrate supplementation can enhance performance and sport-specific tasks by 1–3% in efforts lasting less than 40 minutes [2]. This is because the increase of nitric oxide via dietary NO_3_− helps to improve blood flow [3], oxygen delivery [4], and energy efficiency (i.e. measuring oxidative phosphorylation efficiency) during aerobic exercise [5]. Studies have shown performance improvements of 4% to 25%, particularly in tasks that emphasize time to exhaustion [6], while a >5 mmol of dietary NO_3_− intake has been suggested as a minimum threshold to obtain consistent benefits in exercise performance [7]. A recent meta-analysis conducted by Esen et al. (2023) has established that beetroot juice intake can also induce improvements in short-term anaerobic-based exercise with benefits on peak power output, mean power output, and time to reach peak power output during dynamic exercises with less <10 seconds of duration. This suggests that beetroot juice could be considered as an ergogenic aid for power-based exercise activity, such as a 60 or 100 m sprint [8]. However, no studies have yet examined the impact of beetroot juice supplementation on 60 and 100 m sprint performance. Therefore, investigating its effects on neuromuscular and contractile properties is crucial for understanding its potential benefits in short-distance track and field events.

Although the activation of the NO_3_− to NO_2_− to NO pathway after beetroot juice intake has been primarily linked to mechanisms that enhance oxidative energy production, this pathway is believed to enhance sarcoplasmic reticulum calcium release and reuptake, leading to increased force production in fast-twitch muscle fibers [9]. Additionally, this pathway has been associated with increased neurotransmitter release [10] and enhanced motor unit recruitment [8] all of which could contribute to improved neuromuscular performance in short and explosive exercise activities such as short sprints (i.e. 20 m) or vertical jump capacity [11,12]. The benefits of beetroot juice intake on muscle contraction or neuromuscular parameters are diverse, with evidence of enhanced force, velocity, and power in amateur team-sport athletes and resistance-trained men [13,14] or electromyography activity in healthy untrained men [15]. However, to the best of the authors' knowledge, no previous studies have examined the effects of beetroot juice ingestion on Olympic short sprint disciplines, such as the 100 m sprint, while simultaneously assessing muscle contractile function using measures of involuntary contractile properties, such as tensiomyography.

The aim of this study was first to evaluate the acute (single-dose) effect of beetroot juice ingestion on sprint performance and, second, to assess its effects on vertical jump performance and neuromuscular properties (i.e. via tensiomyography) in trained male sprinters competing in short-distance track and field disciplines. We hypothesized that the ingestion of 70 mL containing 6.4 mmol NO_3_− via beetroot juice would improve sprint performance, expressed with lower 60 and 100 m sprint times while it would enhance neuromuscular function and mechanical properties measured with tensiomyography.

## 2. Methods

### 2.1. Study participants

**Figure 1.**
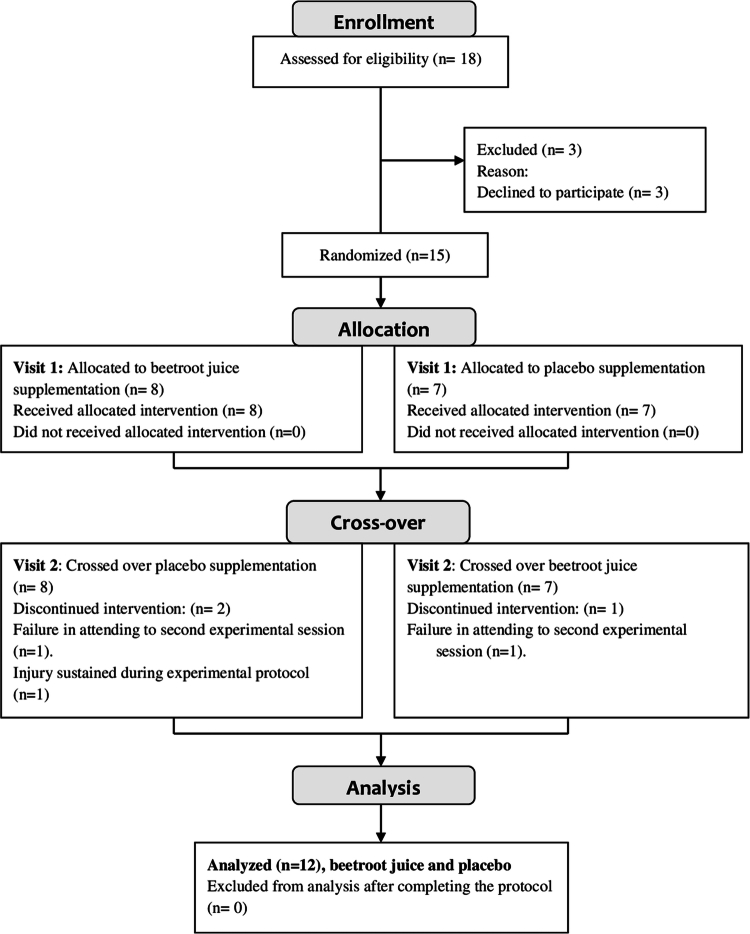
CONSORT flowchart of randomized, double-blind, and crossover experiments.

Initially, 18 male trained athletes from the same competitive track and field club were assessed for eligibility. Inclusion criteria included: (a) age between 18 to 40 years, (b) regular training practice in short-distance track and field disciplines (i.e. 100–400 m) (c) and a minimum of 3 years of athletic training experience (d) participation in regional and national competitions in the year prior to the investigation. Exclusion criteria for participation included: (a) intolerance to beetroot juice or NO_3_− derivatives, (b) presence of any chronic pathology or injury within three months prior to the study, (c) use of medications or supplements (e.g. caffeine) during the study, and (d) failure to attend all experimental sessions and, (e) failure to adhere to the dietary guidelines established in the study. Fifteen participants were recruited for this study after the initial screening. Three athletes were excluded from the analysis: two due to failure to attend the one experimental session (*n* = 2) and one due to an injury sustained during the experimental protocol (*n* = 1; Figure 1). As a result, the final study sample consisted of 12 male sprinters (age: 24 ± 7 years; height: 1.76 ± 0.07 m; body mass: 68.2 ± 6.7 kg; body mass index: 22.14 ± 1.35 kg/m^2^; track and field experience: 11.08 ± 5.50 years; mean training volume: 7.83 ± 3.79 hours/week (classified as Tier 2, trained participants [16]. After being fully informed of the experimental protocols, all participants provided informed written consent to participate. The study was approved by the Bioethics Commission of the Francisco de Vitoria University (approval no: 46/2018), complied with the Declaration of Helsinki, and was retrospectively registered by 1 November 2024 in ClinicalTrials.gov, U.S. National Institutes of Health (Identifier: NCT06675682).

### 2.2. Experimental design

A randomized, double-blind, placebo-controlled crossover experiment was used in this study. An *a priori* sample size calculation using G*Power (version 3.1.9.2; University of Düsseldorf, Düsseldorf, Germany) indicated that a minimum of eight athletes was required to detect statistically significant differences for a matched-pairs t-test analysis, assuming an effect size of 1.20, *β* = 0.80, and two-tailed *α* = 0.05. This calculation was based on a previous investigation that found a benefit of this magnitude when using 4 mmol of NO_3_− via beetroot juice when testing recreationally active males through a countermovement jump [17]. Each athlete participated in two identical experimental trials separated by one week to allow physical recovery between testing and washout. The experimental trials were identical and only differed in the drink ingested before exercise. In one occasion, participants were given 70 mL beetroot juice (6.4 mmol of NO_3_−; Beet-It-Pro Elite Shot, James White Drinks Ltd., Ipswich, UK) made from concentrates of beetroot juice (98%) and lemon juice (2%) (nutritional information (per 100 mL), energy: 373 kJ; fat: 0 g; carbohydrates: 18 g [sugars: 17 g]; protein: 3.7 g; salt: 0.48 g). On a different day, participants were given 70 mL of a taste-matched placebo drink (0.04 mmol of NO_3_−, Beet-It-Pro Elite Shot, James White Drinks Ltd., Ipswich, UK). The drinks were given at same time on each day (16:00 h), in opaque bottles to avoid the identification of the substances ingested and 150 minutes before the start of the performance measurements, following previous protocols [18]. This timing was chosen to align with previous studies that established the peak response nitrate/nitrite (NO_3_− and NO_2_−) occurs 2–3 hours after ingestion [19]. The order of the trials was randomized for each athlete by using a randomization online tool (https://www.randomizer.org/). An independent researcher assigned alphanumeric codes to each trial to ensure blinding of both athletes and researchers during and after the trials. These codes were disclosed to the researchers only after the statistical analysis was completed. Five days prior to the initial testing session, the athletes participated in a familiarization session. This session included detailed explanations, demonstrations, and the completion of the testing battery to reduce potential learning effects. To ensure consistency in test administration and measurement across sessions, all tests were conducted in the same sequence, at the same track and field venue where the athletes regularly trained, and at the same time of day (between 18:00 and 19:00 hours) to minimize the influence of circadian variations on performance [20], during the 2021 competitive season. The testing was carried out midway through the in-season phase. During the testing sessions, the mean ± standard deviation (SD) air temperature was 16.0 ± 1.3 °C, and the relative humidity was 38.5 ± 6.1%, as measured by a portable weather station (Meteorological Station, Küken, Spain).

### 2.3. Experimental protocol

Two days before each experimental trial, dietary nitrate (NO_3_−) intake was restricted by instructing the athletes to avoid nitrate-rich foods, such as beetroot, celery, lettuce, arugula, and spinach, as outlined in written guidelines [21]. Additionally, participants were advised to refrain from brushing their teeth, using oral antiseptic rinses, or chewing gum that could alter their oral microbiota for the 24-hours before the experimental sessions to prevent any potential interference of these routines with the intake of NO_3_− in the experimental trials [22]. In the experimental sessions, a saliva sample was obtained for determining NO_3_− and NO_3_− concentrations 150 minutes after beetroot juice or placebo ingestion (just before the onset of the measurements). The saliva samples were stored at −20 °C until subsequent analysis [23]. On a later day, saliva concentrations were measured using a nitric oxide assay kit (EMSNO K195325, ThermoFisher Scientific, Roskilde, Denmark) following the manufacturer’s instructions and as previously described. All samples were measured in duplicate, and the average values were used for analysis. Participants were also instructed to refrain from any type of exercise 24 hours before testing and to follow a daily energy intake breakdown of 60% carbohydrates, 30% fats, and 10% proteins. All study participants completed 3-day food diaries prior to testing to confirm adherence to the macronutrient recommendations [24]. Upon completion, macronutrient intake was reviewed by a member of the research team to ensure continued adherence to the nutritional guidelines throughout the study. Participants were also instructed to drink 500  mL of water 1–2 hours before arriving at the facility to increase the likelihood of euhydration status (verified by urine specific gravity < 1.020 upon arrival [25].

**Figure 2.**
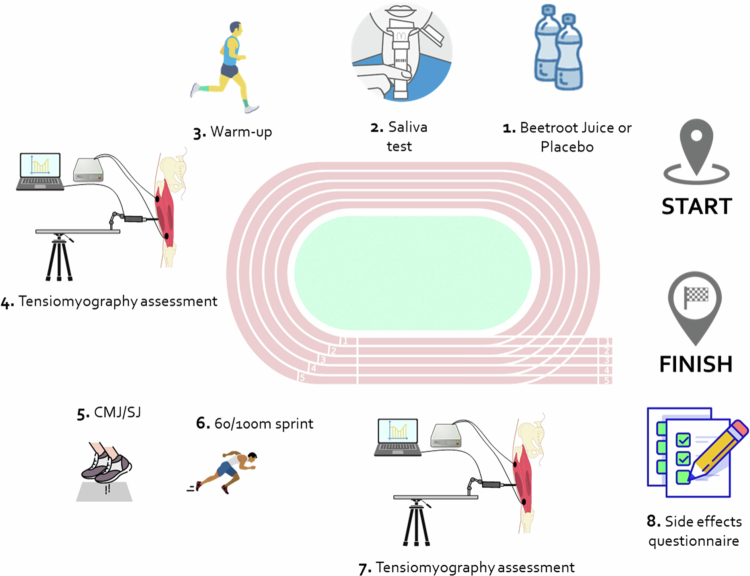
A schematic diagram outlining the sequence of experimental procedures at each testing session. CMJ = countermovement jump. SJ = squat jump.

On the day of the experimental trials, the athletes arrived at their habitual track and field facility and ingested the assigned drink (beetroot juice or placebo juice) while ingestion in its entirety was verified by an experimenter. Then, body mass and body composition were measured using a Tanita B-601 device (Tanita Corp, Tokyo, Japan), and tympanic temperature was recorded with a Thermoscan 7-IRT 6520 (Braun, Frankfurt, Germany) to ensure consistent conditions before each trial. Just after the collection of the saliva sample, participants completed 10 minutes of standardized warm-up (including 5 minutes running at low intensity and 5 minutes of dynamic exercises) and the muscle contractile properties assessment was conducted via tensiomyography on the rectus femoris, biceps femoris, gastrocnemius lateralis, and gastrocnemius medialis of the right leg under standardized conditions. A detailed description of this method is below. Following this tensiomyography assessment, they performed the battery of performance tests. This battery included evaluations of vertical jump capacity through countermovement and squat jumps, as well as 60 and 100 m sprint running tests, as explained below. Twenty minutes after completing the performance tests battery, a second tensiomyography assessment was conducted following the conditions of the pretesting measurement. Additionally, rating of perceived exertion (RPE) was individually assessed for each athlete 30 minutes after completing the entire neuromuscular testing battery in each trial, using an adapted version of the 10-point Borg category-ratio scale [26]. Finally, participants completed a questionnaire following each experimental trial to report any side effects associated with the ingestion of the drinks, using yes/no responses, as employed in previous studies [12,27]. This questionnaire also included a question asking what they believed they had ingested in that specific trial (“beetroot juice”, “placebo” or “I do not know).” The assessment of blinding success was performed using the Bang Blinding Index [28]. A depiction of the experimental protocol can be seen in Figure 2.

### 2.4. Performance tests

Vertical jump capacity was measured in each trial with a countermovement jump and a squat jump. The countermovement jump was initiated from a stationary standing position with weight evenly distributed over both feet, followed by a descent to ~90° knee flexion followed by an explosive jump to reach maximum height. Participants were instructed to keep their hands on their waist throughout the entire countermovement jump [29] and to land on the same location as the takeoff. For the squat jump, participants began from a static position with their knees flexed at a 90° angle and were instructed to avoid any movement to eliminate the contribution of elastic energy on the jump. After 4 seconds in this position, participants performed an explosive concentric movement [30] to reach maximum height, following the arms position described for the prior jump. A researcher verified the execution of each jump and discarded those that did not follow a correct execution either during the takeoff or landing. The jump obtained in each was obtained by using a commercially available jump mat (Chronojump, Boscosystems®, Barcelona, Spain). Two attempts were performed in each jump, with one minute of rest between attempts and types of jumps. The highest height of the two attempts was recorded and used for subsequent statistical analysis. Participants used their training running shoes for these jumps. Verbal feedback was given to encourage athletes to produce maximal performance in each repetition. ICC for jump height in these jumps ranged from 0.97 to 0.98 [31].

After a 5 minutes recovery period, participants completed simulated 60 and 100 m sprint races, with a 15 minutes recovery interval between races. The races were conducted on an official athletic track, with athletes starting from homologated starting blocks. For these simulated competitions, athletes performed their habitual precompetition routines regarding physical and mental preparation. The sprints were initiated by an acoustic signal and running time was measured using two electronic timing sensors (Microgate, Polifemo Radio Light, Italy) positioned at the start and finish of each sprint (60 and 100 m, respectively). All sprints were performed in the central running lane, with no other athletes present on the track during the tests to prevent any potential interference from other runners, as recommended in previous investigations [32]. Participants were allowed to use their spikes for these measurements and researchers verified that the characteristics of the spikes were the same for both experimental trials.

### 2.5. Muscle mechanical properties assessment

Tensiomyography is a noninvasive technique used to assess muscle quality by estimating *in vivo* the contractile and mechanical properties of individual muscles in response to an electrical stimulus [33]. This method quantifies several muscle contractile parameters being the most commonly reported time delay (Td), contraction time (Tc), maximal deformation (Dm), relaxation time (Ts), and sustentation time (Ts) [34]. Since it does not require physical effort from the participants being evaluated, tensiomyography has been employed to objectively measure muscle responses in both non-fatigued [35] and fatigued [36] states. A tensiomyography device (Tensiomyography System 100, Ljubljana, Slovenia) was utilized to assess the contractile properties of the rectus femoris, biceps femoris, gastrocnemius lateralis, and gastrocnemius medialis muscles. All measurements were performed under static and relaxed conditions by a specialized researcher with 10 years of experience in tensiomyography and blinded to the treatments. All measurements adhered to previous recommendations established in the literature [37]. Measurements were taken with the participant in either the supine or prone position, depending on the muscle being assessed, using a standard foam cushion to ensure the muscles were in a relaxed state. Self-adhesive electrodes (4.5 × 4.5 cm) were applied on the skin above muscle bellies, with the cathode positioned proximally to the anode at a distance of 5 cm [34]. A digital displacement transducer (GK 40, Panoptik d.o.o., Ljubljana, Slovenia) was placed between the electrodes using a tripod, positioned perpendicular to the muscle belly to measure deformation at each point with an initial pressure of 0.135 MPa. This pressure was standardized using a reference on the linear transducer. The TMG-S2 stimulator (EMF-FURLAN & Co. d.o.o., Ljubljana, Slovenia; 0–110 mA) delivered an electrical current intensity of 100mA for 1 millisecond (with a range of 0.5–2 ms) at each point. This approach was selected to standardize the number of measurements and to avoid the effects of post-activation potentiation [37,38]. Prior to the stimulation that preceded the measurement of muscle contractile properties, a familiarization stimulation of 40 mA was performed. A 30 s recovery between stimuli was established, following the recommendations raised by previous authors [39]. In the context of tensiomyography parameters, a reduced displacement (Dm) is interpreted as an increase in muscle belly stiffness [36], while an increase of contraction time (Tc) has been considered indicative of muscle fatigue rate [38]. Additionally, delay time (Td) refers to the time between the electrical impulse and the achievement of 10% of maximum contraction, while sustain time (Ts) and relaxation time (Tr) represent the durations between 50% of contraction 370 time to 50% of relaxation time and from 90% of contraction time to 50% of relaxation time, respectively [40]. The Intraclass Correlation Coefficients (ICC) for all analyzed muscle contractile parameters ranged from 0.77 to 0.97 [40].

### 2.6. Statistical analysis

All variables are presented as mean ± SD for the whole group of athletes in each treatment. The normal distribution of the data was confirmed using Shapiro–Wilk tests. Data on performance variables in the placebo *vs* beetroot juice treatments were compared using paired-sample t-tests and Cohen's *d* effect sizes (ES) (±95% confidence intervals). The effect size was interpreted as follows: trivial = 0–0.19, small = 0.20–0.49, medium = 0.50–0.79, and large = > 0.80 [41]. For the contractile properties measured with tensiomyography, a repeated measures MANOVA 5x2x2 (5 parameters × 2 conditions [beetroot juice vs. placebo] × 2 time points [pre vs. post]) was conducted. Partial eta squared (ηp²) was used to report the effect size of the MANOVA, with values below 0.06 considered a small effect, between 0.06 and 0.14 a moderate effect, and above 0.14 a large effect. The McNemar's test was used to detect differences in the prevalence of side effects between conditions. Statistical difference was defined as *p* < 0.050. All statistical analyzes were performed using JASP software version 0.19.0.0 (Amsterdam, The Netherlands), while graphs were created with GraphPad Prism version 8.0.2 (San Diego, United States).

## 3. Results

### 3.1. Salivary NO_3_− and NO_2_− concentrations

In comparison to the placebo, the intake of beetroot juice resulted in statistically significant increases in salivary concentrations of NO_3_− (230 ± 435 vs 6164 ± 3370 μM; *p* = < 0.001; ES = 1.53 [0.58; 2.43] and NO_2_− (130 ± 131 vs 4509 ± 4895 μM; *p* = 0.008; ES = 1.63 [0.65; 2.59]).

### 3.2. Neuromuscular performance tests

**Figure 3.**
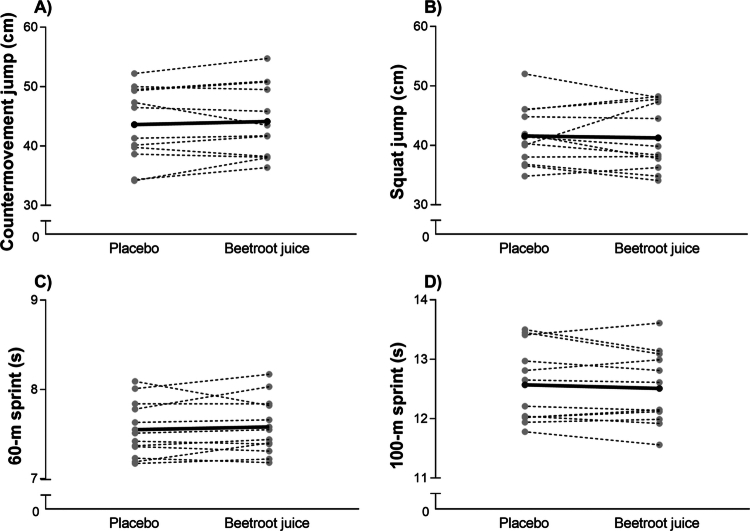
Countermovement jump height A), squat jump height B), 60 m sprint time C), and 100 m sprint time D) with the ingestion of 6.4 mmol of nitrate in the form of beetroot juice or a placebo in male trained sprinters. Means for a group of 12 participants in each treatment are represented by a horizontal black bar and each individual response is represented by a discontinuous gray line.

No differences were observed between the intake of the placebo or the beetroot juice in change in countermovement jump height (*Δ* 1.37%; *p* = 0.400; ES = −0.25 [−0.82; 0.35]; Figure 3a), squat jump height (*Δ* 0.77%; *p* = 0.911; ES = 0.04 [−0.55; 0.62]; Figure 3b), 60 m running time (*Δ* −0.39%; *p* = 0.407; ES = −0.24 [−0.82; 0.33]; Figure 3c) and in 100 m running time (*Δ* 0.47%; *p* = 0.343; ES = 0.29 [−0.30; 0.86]; Figure 3d).

### 3.3. Muscle mechanical properties assessment

The analysis of the mechanical properties of the muscles did not reveal significant interactions between the experimental conditions and the pre-post testing assessments for any of the muscles analyzed: rectus femoris (F(5,5) = 2.034, *p* = 0.227, 1-*β* = 0.299), biceps femoris (F(5,5) = 1.454, *p* = 0.346, 1-*β* = 0.222), gastrocnemius lateralis (F(5,5) = 1.793, *p* = 0.269, 1-*β* = 0.267), and gastrocnemius medialis (F(5,5) = 0.790, *p* = 0.599, 1-*β* = 0.138; Table 1). The only interaction present was for Tr in the biceps femoris (*p* = 0.047; ηp² = 0.37). While the placebo drink increased Tr from the pre-to-post testing assessment, this variable was not modified from the pre-to-post testing in the trial with beetroot juice.

**Table 1.** Tensiomyography variables in the rectus femoris, biceps femoris, gastrocnemius lateralis, and gastrocnemius medialis muscles in the right leg before and after a battery of performance tests with the ingestion of 6.4 mmol of nitrate in the form of beetroot juice or a placebo in male trained sprinters. *Statistically significant differences between treatments at *p* < 0.050.

	Placebo		Beetroot juice			
	Pre	Post	Pre	Post	*p*-value	ηp^2^
RF						
Td (ms)	23.33 ± 2.17	23.1 ± 1.83	24.03 ± 1.96	23.98 ± 1.6	0.823	0.01
Tc (ms)	30.13 ± 5.85	28.12 ± 3.76	29.03 ± 5.68	29.17 ± 4.31	0.358	0.09
Dm (mm)	9.27 ± 2.78	8.91 ± 2.78	8.72 ± 2.21	8.98 ± 1.75	0.637	0.03
Tr (ms)	71.95 ± 64.21	29.69 ± 22.83	40.95 ± 36.28	47.37 ± 60.35	0.209	0.17
Ts (ms)	163.73 ± 163.41	64.52 ± 25.81	75.86 ± 40.24	81.86 ± 59.69	0.109	0.26
BF						
Td (ms)	20.2 ± 1.62	20.21 ± 2.12	20.67 ± 2.06	19.93 ± 1.98	0.336	0.10
Tc (ms)	22.26 ± 6.97	24.2 ± 9.55	23.19 ± 6.74	23.32 ± 9.01	0.702	0.02
Dm (mm)	3.99 ± 2.58	4.47 ± 2.29	4.08 ± 2.35	4.02 ± 2.91	0.455	0.06
Tr (ms)	42.06 ± 39.38	82.9 ± 54.8	84.68 ± 82.06	61.64 ± 43.79	0.047*	0.37
Ts (ms)	224.15 ± 87.55	173.39 ± 42.54	250.93 ± 132.5	160.17 ± 49.4	0.065	0.33
GL						
Td (ms)	19.49 ± 2.06	19.3 ± 1.08	19.49 ± 1.21	19.5 ± 1.16	0.804	0.01
Tc (ms)	19.87 ± 4.19	19.88 ± 1.44	20.51 ± 3.58	19.07 ± 1.83	0.100	0.27
Dm (mm)	3.42 ± 1.32	3.92 ± 1.62	3.24 ± .87	3.7 ± 1.4	0.914	0.01
Tr (ms)	40.33 ± 30.83	46.02 ± 31.68	22.88 ± 7.64	40.22 ± 31.97	0.503	0.05
Ts (ms)	212.74 ± 85.35	158.4 ± 30.63	141.2 ± 54.33	142.02 ± 47.84	0.109	0.26
GM						
Td (ms)	23.5 ± 1.35	23.05 ± 1.4	22.75 ± 3.84	22.89 ± 1.94	0.556	0.04
Tc (ms)	25.52 ± 3.91	23.64 ± 3.01	24.78 ± 6.25	23.75 ± 3.27	0.620	0.03
Dm (mm)	4.53 ± 1.21	5.16 ± 1.61	4.08 ± 2.15	4.24 ± 1.7	0.429	0.07
Tr (ms)	76.77 ± 40.65	76.31 ± 37.06	65.14 ± 41.28	58.72 ± 29.19	0.773	0.01
Ts (ms)	172.05 ± 68.78	150.63 ± 38.5	151.55 ± 20.98	143.65 ± 31.81	0.615	0.03

Abbreviations: RF: Rectus Femoris; BF: Biceps Femoris; GL: Gastrocnemius Lateralis; GM: Gastrocnemius Medialis; Td: Delay Time; Tc: Contraction Time; Dm: Maximal Deformation; Tr: Relaxation Time; Ts: Sustentation Time; ms: milliseconds; mm (millimeters); *p*: *p*-value; ηp² = partial eta squared. Results are expressed as mean ± standard deviation. *statistical differences *p *< 0.05.

### 3.4. Rating of perception exertion and side effects

At the end of the experimental trials, the RPE was similar for the placebo and beetroot juice (3.9 ± 0.8 vs 4.2 ± 1.2 points; *p* = 0.337; ES = −0.24 [−0.78; 0.35]). Additionally, there were no differences in the prevalence of side effects between conditions (*p* = 0.132–1.000; Table 2) were reported. The Bang index was 0.16 for the placebo trial and 0.08 for the beetroot juice trial. These data points toward a “near-to-perfect” blinding for the experiment in both trials.

**Table 2.** Prevalence of side effects reported after beetroot juice or placebo ingestion in male trained sprinters.

Side effect	Condition (%)		*p*-value
	*Placebo*	*Beetroot juice*	
Gastrointestinal problems	8.3	33.3	0.132
Red urine	16.6	16.6	1.000
Gastroesophageal reflux	0.0	8.3	0.307
Nausea	0.0	8.3	0.317
Muscular pain	0.0	0.0	1.000
Headache	16.6	16.6	1.000
Increased urination	16.6	25.0	0.615
Fatigue	0.0	8.3	0.307
Nervousness	0.0	0.0	1.000

Data represents the percentage of athletes who reported the side effect on a yes/no scale from 12 athletes.

## 4. Discussion

The aim of this study was to evaluate the acute effects of beetroot juice ingestion (70 mL with a total of 6.4  mmol of NO_3_−) on sprint performance (60 and 100 m) and neuromuscular performance in male trained sprinters. To the best of our knowledge, this is the first study to determine the effect of beetroot juice intake on 60 and 100 m sprint times and on muscle contractile properties (i.e. measured *in vivo using* tensiomyography). The main outcomes revealed no significant benefits on sprint performance, on jump performance, or in the muscle contractile properties after an acute beetroot juice ingestion compared to the placebo in male trained sprinters. The lack of any performance benefits was present even with abrupt increases in salivary concentrations of NO_3_− and NO_2_−, confirming that the beetroot juice ingestion protocol induced the processing of the nitrates from the juice into nitrite, which is an essential step in the nitrate-nitrite-nitric oxide (NO) pathway. Salivary concentrations of NO_3_− and NO_2_− suggest that the ingested nitrates were being effectively absorbed and prepared for further conversion to nitric oxide (NO). Therefore, although there is some evidence suggesting that acute beetroot supplementation may be considered as an ergogenic aid for short-term all-out exercise activities, the present study refutes this notion, at least for sprint events up to 100 m. Further evidence should address if higher doses or chronic ingestion of beetroot juice may induce an ergogenic effect, but the outcomes of this study clearly reflected a lack of performance-enhancing activity of beetroot juice when ingested acutely and at a dose of 6.4  mmol of NO_3_−.

The measurement of vertical jump height is commonly utilized in sports sciences to evaluate muscle strength and power of the lower limbs [42]. Additionally, strong correlations have been found between the 100 m sprint time and vertical jump capacity (i.e. measured with squat jump countermovement jump) [43] suggesting that vertical jump capacity can be associated to 100 m sprint performance outcomes. According to our data, acute beetroot juice ingestion did not result in an improvement in jump height during single countermovement and squat jumps. Our findings are consistent with previous studies that also did not report any enhancement in vertical jump height in a nonfatigued state in participants who were ingesting beetroot juice acutely [18,27,44]. Therefore, future studies should be conducted to analyze additional metrics of vertical jump performance beyond just vertical jump height (e.g. peak power and mean propulsive force) [45]. This may provide a more accurate reflection of muscle contractile changes induced by acute beetroot juice ingestion and could provide a more comprehensive evaluation of any potential effectiveness of this substance.

In the same line, the present study demonstrated that beetroot juice ingestion did not significantly improve 60 and 100 m sprint times in trained male sprinters, with mean differences ranging from 0.03% to 0.06%. To the authors' knowledge, this is the first study in the literature to investigate this specific topic, and comparison with previous studies is challenging; however, there is a particularity of this type of exercise that may have limited the benefits of acute beetroot juice ingestion. Acute beetroot juice supplementation appears to exert its ergogenic effects predominantly under conditions of elevated metabolic stress, such as repeated-sprint exercise or hypoxic and acidotic environments, where phosphocreatine availability is reduced, and force production becomes progressively impaired [46,47]. Under these conditions, beetroot juice has been shown to enhance muscle contractile efficiency, potentially through improved motor unit recruitment and more efficient calcium handling at the muscular level, mechanisms that are particularly relevant during the development of neuromuscular fatigue [48]. Consequently, when exercise tasks are performed in a non-fatigue state, such as isolated, maximal sprints, the physiological constraints that beetroot juice supplementation is proposed to alleviate are largely absent, which may explain the limited ergogenic effects observed in such contexts. Furthermore, future research should focus on evaluating the potential efficacy of beetroot juice supplementation during the different phases of a 100 m sprint (i.e. block start and acceleration, maximum velocity and deceleration phases) [49], given that beetroot juice has been shown to reduce the time to reach peak power output during a Wingate cycle sprint test [50].

A novel aspect of our study was the application of tensiomyography to evaluate the effects of beetroot juice on muscle contractile properties in trained male sprinters, in a nonfatigued state (i.e. prior to a neuromuscular test battery) and in a fatigued state (i.e. after a series of neuromuscular efforts). Previous studies have reported that tensiomyography assessment is able to detect changes in muscle properties induced by other dietary supplements such as caffeine [51] or multiingredient beverage (i.e. mixture of carbohydrates and protein combination) [52] suggesting the convenience of this technique to assess muscle properties changes in experiments testing the mechanisms associated with ergogenic aids. Although the ingestion of beetroot juice has been associated with enhancing some contraction mechanisms in type II (i.e. fast-twitch) muscle fibers [53], our study did not report changes in tensiomyography variables between beetroot juice ingestion and the placebo, except for a marginal effect on Tr in the biceps femoris that was not present in the remaining muscles tested. This was somewhat expected in the light of previous results of this study, as the lack of any sprint or jump performance benefit with beetroot juice ingestion aligns with the lack of benefits at the muscle level. Overall, the current investigation confirms a lack of performance benefit of acute beetroot ingestion on sprint performance associated with the absence of changes in the muscle properties of the muscle measured by tensiomyography.

Despite its strengths, the current study has several limitations that should be discussed to enhance its relevance to real-world sports contexts. First, we only investigated the effects of an acute supplementation protocol using 70 mL beetroot juice containing 6.4 mmol of nitrate (NO_3_−) ingested 150 minutes before the onset of performance testing. This same dosage and timing protocol has been employed in other investigations that found an ergogenic benefit of beetroot juice ingestion [50]. However, different dosages or timings for beetroot juice supplementation may produce varying effects on muscle contractile properties and neuromuscular performance [12,14]. Further research is required to determine if an optimal supplementation regime exists for sprinters, perhaps with the inclusion of higher doses or using chronic supplementation protocols for beetroot juice. Second, while this study focused on male-trained sprinters, more studies with broader samples that include female athletes and athletes at different competition levels are recommended to strengthen the evidence base on this topic, given that factors such as sex and training status [54] may influence the response to beetroot juice supplementation. Third, our testing included the measurement of sprint times during simulated 60 and 100 m races to enhance the ecological validity of the study. However, the measurements were made in a noncompetitive environment with only one participant tested individually. Future studies should aim to measure the ergogenic properties of beetroot juice supplementation during official competitions to enhance the ecological validity of the results further. Fourth, plasma NO_3_− and NO_3_− concentrations could not be obtained; therefore, salivary samples were used to assess nitrate and nitrite availability.

## 5. Conclusion

Acute ingestion of 70 mL beetroot juice containing 6.4  mmol of NO_3_− did not enhance sprint performance and neuromuscular function of male trained sprinters. The outcomes of this study confirmed that beetroot juice supplementation did not modify power as measured via jump performance, 60 and 100 m sprint times, and did not modify any muscular property measured in key muscles for propulsion. This supplement should not be considered as an ergogenic aid for track and field sprinters.
